# Caring for Others, but Not Themselves: Implications for Health Care Interventions in Women with Cardiovascular Disease

**DOI:** 10.1155/2011/376020

**Published:** 2011-05-22

**Authors:** Michelle DiGiacomo, Patricia M. Davidson, Robert Zecchin, Kate Lamb, John Daly

**Affiliations:** ^1^Centre for Cardiovascular and Chronic Care, Faculty of Nursing, Midwifery and Health, University of Technology Sydney and Curtin University, P.O. Box 123, Broadway, NSW 2007, Australia; ^2^Centre for Cardiovascular and Chronic Care, Faculty of Nursing, Midwifery and Health, University of Technology Sydney, Broadway, NSW 2007, Australia; ^3^Area Cardiac Rehabilitation and Chronic Care Programs, Sydney West Area Health Service, Westmead Hospital, Westmead, NSW 2145, Australia; ^4^Clinical Support Division, Western Sydney Local Health District, LMB 7118, Parramatta NSW 2150, Australia; ^5^Faculty of Nursing, Midwifery and Health, University of Technology Sydney, P.O. Box 222, Lindfield NSW 2070, Australia

## Abstract

Cardiovascular disease is the largest killer of women internationally and women often suffer inferior outcomes following an acute cardiac event as compared to men. A gendered approach to investigating cardiovascular disease in women incorporates the unique social, cultural, and economic circumstances that being a woman brings to the health encounter. The multiple roles enacted by many women may be important factors in this health discrepancy. In order to more fully understand the impact of the roles of women on health, a questionnaire was administered to participants of the *Heart Awareness for Women* group cardiac rehabilitation program which assessed women's role perceptions followed by discussions. We found that caregiving can be both positive and negative. It gives a sense of purpose, meaning, and community connection as well as burden and conflict. Emphasis must be placed on promoting strategies in women to achieve a balance between caregiving responsibilities and prioritisation of cardiovascular health.

## 1. Introduction

Cardiovascular disease (CVD) is the largest killer of women internationally [1]. Women often suffer inferior outcomes following an acute cardiac event as compared to men and are often diagnosed less promptly and treated less aggressively [2]. Fewer women attend cardiac rehabilitation (CR) than men [3], despite its proven benefits to functioning and psychosocial wellbeing [4]. 

The disparity in health outcomes between men and women in cardiovascular disease is contested. It has been argued that it is the older age at which women get CVD rather than gender issues that contribute to their worse outcomes. However, it is impossible to discuss the context of women living with heart disease without considering a gendered approach. According to Williams and Kurina [5], of all the social determinants of health, gender is one of the most significant. A gendered approach considers the unique social, cultural, and economic circumstances that being a woman brings to the health encounter. A focus on the reproductive health care needs of women is still prominent and there is a disregard for women's health problems that still may be evident today. Women who have CVD are generally older and sicker than their male counterparts. They have more hypertension, diabetes, longer ICU stays, and poorer outcomes. This may be related to problems with referrals, women's experiences, or their perceptions of themselves and their illness [6].

Numerous studies suggest that women's inferior health outcomes exist because women do not prioritise their own personal health needs. Delaying seeking help for symptoms can have catastrophic implications for heart disease as there is an inverse relationship between time to accessing medical treatment at the onset of symptoms and health outcomes [7]. Despite this common assertion, few studies have attempted to identify reasons for women not prioritising their health, and as a consequence, there are fewer interventions targeting women specifically. The success of the *Go Red for Women* campaigns confers some optimism that if interventions are developed for women using a gendered approach within a social marketing framework, alterations in women's experience in the illness trajectory of heart disease can be achieved [8].

The multiple roles enacted by many women, including wife, employee, and mother, are broadly considered beneficial to their health; yet this is potentially mediated by socioeconomic circumstances [9]. Particularly for women experiencing socioeconomic deprivation or who are single parents, multiple roles have been hypothesized as detrimental to health and wellbeing [10]. In addition to the aforementioned roles, women are twice as likely as men to assume unpaid caregiving responsibilities for young children, the elderly, the disabled, and mentally and physically ill family members and spouses and often this is in addition to other paid works [11, 12]. 

Caregiving takes a toll on health and wellbeing, the effects of which can compound the disproportionate incidence of various chronic diseases in women [13]. In a national representative survey of women's health in the USA, one in four women caregivers rated themselves as being in poor or fair health. More than half of these women had one or more chronic conditions compared to approximately 41% of the noncaregiver women [12]. Women caregivers were also twice as likely to report a time in the past year when they did not get needed medical care in that time [12, 14]. Furthermore, Schulz and Beach [15] found that being an older caregiver who is experiencing mental or emotional strain is an independent risk factor for mortality. 

Caregivers can be especially vulnerable in that they are significantly more likely to be in poor health and are more likely to report having difficulties getting needed medical care [12]. The stress of caregiving can render caregivers more susceptible to illness [13] and health complications than other women [12]. Despite interacting with doctors and other medical professionals in a caregiver capacity, caregivers are less likely than other women to perceive and report difficulties in accessing medical care for themselves [12].

Roles enacted by women entail demands on time and psychological, emotional, physical, and financial resources. These demands are compounded with each additional role undertaken. Feelings of stress and being overwhelmed can result from an inability or a perceived inability to meet these demands and, as such, can tax coping skills [16]. Stress reactions can instigate physiological and behavioural changes that can increase risks for cardiovascular disease. Attempting to fulfill these roles may impact on whether and how women attend to their own health including getting enough sleep, exercise, good nutrition, respite, and psychological and emotional support. Stress and inadequate coping may contribute to development of depression and cardiovascular disease. Depression itself is a risk factor for cardiovascular disease [17] and is associated with physiological and behavioural changes. Caregivers have been found to have significantly higher levels of depression and stress, and lower levels of general subjective wellbeing than noncaregivers [18], particularly in women [19]. Given that many female caregivers also have paid jobs, the strong evidence of job strain as a risk factor for cardiovascular disease in women suggests further detriment [20, 21]. Furthermore, excessive household and family demands and low control at home predict coronary heart disease in women [22].

For CR programs to be successful, they must consider these issues that women face. In order to more fully understand the impact of the roles of women and how these impact on health, a questionnaire assessing women's role perceptions was administered to participants of the Heart Awareness for Women Program (HAFW) [23]. Briefly, the HAFW program was a nurse-facilitated mutual aid model CR program in which groups of 5–10 women met weekly for 6 weeks to learn about and discuss CVD in women, strategies for behavior change, social support, and coping with stress and change. The aim of this paper is to describe women's perceptions and experiences with caregiving and the different roles that comprise their lives in order to assist them to engage in strategies to address their health care needs.

## 2. Materials and Methods

Women participating in a group CR program tailored to the needs of women [23] at two hospitals in Western Sydney, Australia, were asked to complete the Women's Role Inventory Protocol-Modified questionnaire as well as participate in group discussions. The majority of these women had had an acute coronary syndrome event while a minority had coronary artery bypass grafting and chronic heart failure. The Women's Role Interview Protocol (WRIP) [24] was developed to determine the degree of stress and satisfaction derived from five traditional female gender roles including wife/partner, mother, housework, caregiver, and paid employee. For each role, women were asked to rate their level of stress and satisfaction along a 10-point scale. The stress ratings for each role ranged from 1 (not stressful at all) to 10 (very stressful), and the satisfaction ratings for each role ranged from 1 (not satisfying at all) to 10 (very satisfying). 

The WRIP-Modified was used to facilitate group discussion about these issues as well as to guide the exploration of the impact of roles on health-seeking behaviours. We assessed the impact of traditional gender roles on women's recovery from an acute cardiac event and their capacity to address cardiovascular risk factor modification. Group discussion enabled women to freely comment on these topics to provide contextual information not captured by questionnaire items. Participants provided rationale for their ratings, often sharing examples from their daily lives. Fieldnotes containing dialogue and observations were recorded by two researchers facilitating the groups. The first level of analysis involved the two researchers separately interpreting proceedings by reading and note-taking. The researchers then met to explore, debate, and synthesize their interpretations. During the final stage of analysis, themes which emerged from the qualitative findings were considered in light of quantitative results. Approval to undertake this study was granted by the Area Health Service Human Research Ethics Committee and informed consent was obtained from all participants. 

## 3. Results and Discussion

Fifty-four women enrolled in the program and took part in discussions. Of those, 45 participants completed the WRIP-Modified. The mean age of participants was 60.85 years (SD 9.1) with a range of 42–80 years and the majority (71%; *n* = 32) were married and living with their spouse or intimate partner. Among the remainder of participants, 11% (*n* = 5) were divorced, 20% (*n* = 9) were widowed, and 14% (*n* = 6) were living alone. Most participants had no children living at home, but 18% (*n* = 8) had between 1 and 4 children, ranging in age from 1 to 48 years old, living with them. Eleven percent (*n* = 5) of participants were caregivers for people living outside their homes. Participants in paid employment comprised 16% (*n* = 7) of the sample and a further 7% (*n* = 3) held volunteer positions in various organizations. 

**Figure 1:**
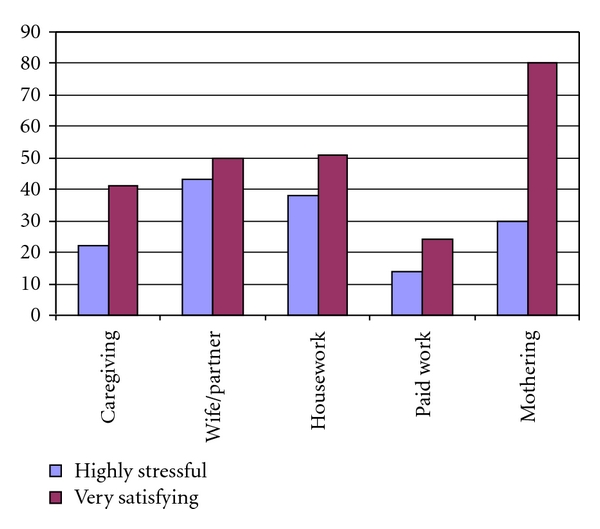
Results of WRIP-Modified (*n* = 45).

As depicted in Figure 1, participants rated each of the five domains of the WRIP-Modified as more satisfying than stressful. Despite marked differences in perceptions of satisfaction and stress for the majority of roles, the spousal role was described as almost equally stressful and satisfying, a finding similar to that reported by Meleis and Stevens [25]. Alternatively, motherhood was perceived as much more satisfying than stressful and potentially highlights an area of further research. This was in contrast to the paid work role for which less satisfaction was reported. Although these domains were stressful to an extent, they also were satisfying, indicating that conceptualization of caregiving roles as always burdensome to women is inaccurate.

The WRIP domain of “caregiving” received particular attention in discussions. “Caregiving” is often described in academic literature as informal, unpaid care provided to address an individual's health needs or to support daily living activities. Participants in this study described their meaning of “caregiving” in broader terms. For them, caregiving was enacted within each of the five domains. They provided care to children and grandchildren in their maternal role, their husbands in their spousal role, their family/household in their housework role, infirmed family and friends (out of household) in the “caregiver” role, and family/household in their employment role by contributing monetary resources. Therefore, “caregiving” was not just about caring for a sick relative but rather was a part of every role they enacted in their lives. 

Psychological benefits were the primary outcomes of these roles. Participants valued and appreciated the connections these roles engendered with friends and families, the feeling of being needed and being useful, and the sense of purpose and meaning in their lives. One woman described how doing household chores contributed to her self-esteem:

*I know I do not have to do all that stuff, like cleaning windows…but you know, it makes me feel good about myself.*

Engaging in these multifaceted caregiving roles supported and contributed to some of the women's self-esteem, self-concept, identity, and social ties. This finding supports the multiple attachment hypothesis which asserts that these roles provide attachment to the community, a likely benefit to women's health [26, 27].

Alternatively, employment status of women and inequality in division of household tasks have been described as stressors in previous research [25]. One participant depicted how her husband's attempts to alleviate what he perceived as strain from her daily life resulted in feelings of loss and despair. Following her heart attack, her husband insisted that he will do all the cooking and cleaning, activities that she previously enacted in the household:

*He would not let me do anything. I just sat there, it was awful. I felt so useless.*

Although potentially well-intentioned, limiting his wife's activities had negative psychological consequences for her. She went on to describe being tearful, despairing, and depressed during this period. Approximately 15–20% of patients have depression following a coronary event and this impacts on their quality of life and engagement in CR and other behavior change activities [28]. Maintenance of identity and self-concept is important to psychological health. Instances such as these contribute to our understanding of the complexities of the spousal relationship, particularly following this life event. These findings have implications for health professional communication with not only patients but also their spouses and families regarding how best to support their loved one following this event and how to promote communication of needs within this partnership. 

Difficulties of managing multiple roles and incorporating advised exercise and stress minimisation strategies were discussed. Some women reported that they either did not attend or missed CR sessions due to caregiving activities or their concomitant effects of emotional or physical exhaustion [23]. Although motherhood was described by some participants as rewarding in the form of reciprocated love and affection and a sense of satisfaction and connectedness, the following excerpts from different women depict less desirable outcomes:

*I love taking care of my grandchildren and helping my kids get on their feet, but I get so tired.*

*They just expect me to be there…“mum will always do it.”*

*Sometimes all their worries [children and family] just drag me down.*

In contrast to the previously discussed limiting of activities by family, more common were statements depicting the enduring expectations of children and family and the toll it takes as women continued to fulfill these roles, despite fatigue. Women often prioritize the needs of their family before their own [23]. Although giving support was apparently second nature to the participants, asking for help was perceived as more difficult. Some participants described not wanting to add to family members' stress by disclosing physical symptoms or psychological burdens [23]:

*I did not want to worry anyone.*

Delaying seeking treatment for symptoms that may be related to CVD or “waiting to see” if symptoms will dissipate unassisted is dangerous and can result in irreparable damage or death [7]. Several participants shared similar statements depicting caregiving roles as barriers to CVD management and risk factor modification, again highlighting implications for health professionals to intervene by raising awareness and assisting women to understand that only by taking care of themselves, they can take care of their families. 

## 4. Strengths and Limitations

A number of caveats have to be considered when interpreting these data. The small, convenience sample and the limited psychometric validation of the WRIP are limitations of this study. The instrument, however, was used not only to garner quantifiable levels of stress and satisfaction but also to extrapolate meaning and context of such perceptions. This study is one of the few studies that has tried to unpack the notion of juggling multiple roles and discriminating between the positive and negative impacts of the caregiving role. These data are useful in interpreting the conceptualization of the care construct.

## 5. Conclusions

The burden of caregiving roles for women underscores the importance of addressing this factor in primary, secondary, and tertiary cardiovascular prevention strategies. In spite of the plethora of data relating to the negative effects of the caregiving role in women, there is minimal data identifying solutions. In addition, there is commonly an emphasis on the negative impacts of caregiving, as reflected in instruments such as the Caregiver Strain Index [29], so there is a need to develop better ways to conceptualise the construct, and to do so, we need to obtain women's perspectives. On the basis of our work, we have identified that caregiving can be both positive and negative. It gives a sense of purpose, meaning and community connection as well as burden and conflict. Our intervention emphasized to women that without caring for themselves, their ability and capacity to undertake their caregiving role would be impaired, and therefore self-care enables caregiving. Women found that taking the time to address their own needs helped them achieve a sense of balance with their multiple roles. In many areas of women's lives where they experience transition in roles, for example, motherhood and menopause, women derive benefit from connecting and communicating with other women. We have found that in parallel with other settings, support from other women can assist in balancing the positive and negative aspects of caregiving. Until recently, heart disease has been perceived as a man's disease and cardiovascular care has commonly focused on the needs of men. Emphasis must be placed on promoting strategies in women to achieve a balance between caregiving responsibilities and prioritisation of cardiovascular health, particularly as the altered population dynamics of ageing mean that women will comprise a higher proportion of the patient population for cardiology health professionals.
